# Persistent multispecies dissemination of *armA*-carrying IncR plasmids among clinical and environmental bacterial populations in a Spanish veterinary hospital

**DOI:** 10.1128/jcm.00673-25

**Published:** 2025-10-09

**Authors:** Carlos Serna, Mario Pulido-Vadillo, Bosco R. Matamoros, Javier F. Favieres, Natalia Montero, Claudia García Berdún, Marta E. García, Jose L. Blanco, Jose F. Delgado-Blas, Bruno Gonzalez-Zorn

**Affiliations:** 1 Antimicrobial Resistance Unit (ARU), Department of Animal Health, VISAVET Health Surveillance Centre, Faculty of Veterinary Medicine, Complutense University of Madrid 16734, Madrid, Spain; 2 Department of Animal Health, Faculty of Veterinary Medicine, Complutense University of Madrid 16734, Madrid, Spain; 3 Veterinary Teaching Hospital, Complutense University of Madrid 16734, Madrid, Spain; University of California, Davis, Davis, California, USA

**Keywords:** veterinary hospital environment, plasmid epidemiology, aminoglycoside resistance

## Abstract

Aminoglycoside resistance mediated by 16S rRNA methyltransferases poses a growing threat in both human and veterinary medicine. Here, we conducted a retrospective genomic study of Enterobacterales isolates (*n* = 789) collected from clinical samples at a veterinary teaching hospital in Spain between 2011 and 2020. We identified four high-level aminoglycoside-resistant ST171 *Enterobacter hormaechei* subsp. *xiangfangensis* isolates carrying the *armA* gene, all from horses. Using Illumina and Nanopore sequencing, we determined that *armA* was located on a 70 kb IncR plasmid, also carrying other resistance genes such as *msr(E*), *mph(E*), *bla*_DHA-1_, and *qnrB4*, embedded within a Tn*1548*-like element. Genomic comparisons indicated that the IncR plasmid was linked to a 2008–2010 ST11 *Klebsiella pneumoniae* outbreak in companion animals at the same hospital, with >95% sequence plasmid identity. Although the IncR plasmid lacked conjugative genes, it was mobilizable *in vitro* via co-resident conjugative plasmids. To probe for an environmental reservoir, we sampled three horse stalls in 2022 and recovered 19 *armA*-positive isolates (*E. hormaechei*, *Klebsiella pneumoniae,* and *Mixta calida*) whose IncR plasmids were nearly identical to that found in clinical clones. Broader analysis of 1,330 IncR plasmids from the genomic plasmid database PLSDB revealed that most were mobilizable, frequently co-integrated with other replicons, and carried diverse resistance genes, though *armA* was uncommon. These findings demonstrate that non-conjugative IncR plasmids can persist in the environment and be horizontally disseminated to clinical isolates over a long period of time, underscoring the need for routine, plasmid-focused genomic surveillance in veterinary healthcare settings within a One Health framework.

**IMPORTANCE:**

The spread of antimicrobial resistance threatens both human and animal health. In veterinary hospitals, bacteria can share resistance genes not only through direct transmission but also via mobile plasmids that persist in the environment. In this study, we uncovered a decade-long persistence of a non-conjugative IncR plasmid carrying the *armA* gene, which confers high-level aminoglycoside resistance, in a Spanish veterinary teaching hospital. This plasmid was found in clinical isolates of *Enterobacter hormaechei*, *Klebsiella pneumoniae,* and *Mixta calida* from horses and from the hospital environment. Our findings show that even plasmids lacking self-transfer capability can be maintained and disseminated across bacterial species over many years. These results highlight the need for routine genomic surveillance of plasmids in veterinary healthcare settings to prevent long-term environmental reservoirs from fueling recurrent outbreaks.

## INTRODUCTION

Aminoglycosides are a long-standing class of antimicrobials that remain essential in contemporary clinical practice. These antibiotics disrupt bacterial protein synthesis by binding to the 30S ribosomal subunit and are effective against a broad range of bacteria, including both Gram-positive and Gram-negative species, with notable activity against *Enterobacteriaceae* (1). In veterinary medicine, aminoglycosides are used to treat infections in various food-producing and companion animals (2). The World Organisation for Animal Health classifies them as critically important veterinary antimicrobials (3), while the World Health Organization designates them as critically important antimicrobials for human medicine (4). Despite their utility, the effectiveness of aminoglycosides has been compromised by the emergence of 16S rRNA methyltransferases (16S-RMTases). These enzymes confer high-level, broad-spectrum resistance to aminoglycosides by methylating the ribosome, thereby preventing antibiotic binding (5). To date, 13 16S-RMTase genes (*armA*, *rmtA-I*, and *npmA-C*) have been identified in Gram-negative and Gram-positive bacteria (5–7).

A critical concern with 16S-RMTase genes is their location on transferable plasmids (e.g., IncA/C, IncI1, IncR, IncN, and IncF) and/or their association with mobile genetic elements like transposons, integrons, and insertion sequences (e.g., Tn*1548* and *armA* or IS*Ecp1* and *rmtC*). These associations facilitate the horizontal spread of antibiotic resistance genes (ARGs) among different bacterial strains and species (8, 9). This horizontal gene transfer is especially problematic in clinical settings. For example, studies have reported the presence of 16S-RMTase genes in *Escherichia coli* strains across hospitals in France, primarily carried on a wide variety of plasmids (10). Similarly, the recent identification of the *rmtE4* variant on an IncL plasmid in hospital environments in America underscores the global nature of this issue (11).

Whole-genome sequencing (WGS) is a powerful tool for uncovering the genetic mechanisms of resistance and tracking the dissemination of resistant strains. While these techniques are increasingly implemented in human healthcare settings (12), their use in veterinary hospitals remains less frequent. Notable findings in veterinary hospital contexts, such as the colonization of handwashing sinks with *Enterobacter hormaechei* carrying multiple ARGs on an IncHI2 plasmid (13) or the dissemination of a *Klebsiella pneumoniae* clone producing *bla*_DHA-1_ on an IncR plasmid over several years in a veterinary setting (14), highlight the need for a more widespread application of WGS in these environments.

Whether 16S-RMTase plasmids can persist and spread over long periods within veterinary hospitals remains unknown. Here, we conducted a retrospective WGS analysis of Enterobacterales isolates collected between 2011 and 2020 at a tertiary veterinary teaching hospital in Spain. We show that a successful IncR plasmid carrying *armA* persisted for at least 10 years, underscoring the need for genomic surveillance and strengthened infection-control strategies in veterinary medicine.

## MATERIALS AND METHODS

### Study setting and clinical bacterial isolates

A retrospective analysis was conducted on bacterial isolates exhibiting phenotypic resistance to aminoglycoside antibiotics, collected from clinical samples at the Veterinary Teaching Hospital of Complutense University of Madrid between 2011 and 2020. During this period, 5,149 bacterial isolates recovered from clinical specimens underwent bacteriological analysis at the hospital’s microbiology unit. The resistance profiles of these bacterial strains were determined using the Kirby-Bauer disk diffusion method (DDM), following the guidelines of the European Committee on Antimicrobial Susceptibility Testing (EUCAST) (15). To identify isolates potentially harboring 16S-RMTase genes, inclusion criteria required that the strains were identified as Enterobacterales via the VITEK 2 system (BioMérieux, France) and exhibited disk inhibition zones of <18 mm for amikacin, <17 mm for gentamicin, and <16 mm for tobramycin, as measured by DDM. Isolates meeting these criteria were included even if only one or two aminoglycosides were tested. Selected isolates were then cultured on Luria-Bertani (LB) agar supplemented with 200 mg/L gentamicin and 200 mg/L amikacin (Sigma-Aldrich Inc., USA) (16) to select for aminoglycoside-resistant 16S-RMTase producers for further investigation.

### Environmental sampling

Environmental samples were collected from the large animal hospitalization area of the Veterinary Teaching Hospital in May–June 2022. Sampling was conducted in three horse hospitalization stalls, specifically targeting high-touch and high-risk surfaces, including the sink, floor, feeder, and manure collector in each stall. For the sampling, 3M sponges (3M Corporation, USA), pre-hydrated with 10 mL of peptone water, were used. A sterile technique was employed to swab a standardized 100 cm^2^ area on each target surface. After sampling, an antibiotic enrichment step was performed by adding 60 mL of peptone water to the sterile bag containing the sponge and supplementing it with 50 mg/L amikacin and 50 mg/L gentamicin. The samples were then incubated at 37°C for 24 hours with agitation. Following enrichment, 100 µL aliquots of the incubated liquid were cultured on MacConkey agar plates supplemented with 200 mg/L gentamicin and 200 mg/L amikacin. The plates were then incubated at 37°C for 24 hours. Colonies with different morphologies from each surface were selected for further analysis.

### Bacterial identification, antimicrobial susceptibility, and 16S-RMTase detection

Bacterial species were identified using matrix-assisted laser desorption/ionization time-of-flight mass spectrometry at the Centro de Vigilancia Sanitaria Veterinaria (VISAVET Health Surveillance Centre, Spain). For isolates identified from the retrospective analysis conducted between 2011 and 2020, minimum inhibitory concentrations (MICs) of various antimicrobial classes were evaluated using the broth microdilution method with commercial Sensititre EUVSEC plates (Thermo Fisher Scientific, USA), following the manufacturer’s specifications. Results were interpreted according to EUCAST guidelines, and when EUCAST guidelines were unavailable, Clinical and Laboratory Standards Institute (CLSI) guidelines were used (17). Additionally, MICs for amikacin, apramycin, gentamicin, kanamycin, and neomycin were determined by in-house broth microdilution assays in accordance with EUCAST guidelines. For the environmental samples collected in 2022, the resistance profiles of the bacterial strains were characterized using the Kirby-Bauer DDM according to EUCAST guidelines. The antibiotics tested included ceftriaxone (30 µg), ciprofloxacin (5 µg), tetracycline (30 µg), ampicillin (10 µg), and imipenem (10 µg). To detect the presence of 16S-RMTase genes (*armA*, *rmtA*, *rmtB*, *rmtC*, *rmtD*, *rmtE*, *rmtF*, *rmtG*, *rmtH,* and *npmA*) in all isolates, PCRs were performed using previously described primers and conditions (18).

### DNA extraction, whole-genome sequencing, and data processing

Short-read Illumina sequencing was performed on all 30 confirmed 16S-RMTase-carrying isolates (4 clinical and 26 environmental) at the Instituto Tecnológico Agrario de Castilla y León (ITACYL, Spain). For each isolate, a single colony was grown overnight in Brain Heart Infusion broth at 37°C. DNA extraction and purification were carried out using the DNeasy Blood & Tissue Kit (Qiagen, Germany) according to the manufacturer’s protocol. DNA concentration was assessed using Qubit (Invitrogen, USA). For a subset of isolates (*n* = 17), long-read sequencing was conducted using Nanopore technology. The sequencing was performed with v14 library preparation chemistry and an R10.4.1 flow cell by the provider Plasmidsaurus’ bacterial genome sequencing service.

The quality of raw sequence data was assessed using FastQC version 0.11.9 (https://github.com/s-andrews/FastQC) for Illumina reads and NanoQC for Nanopore reads (19). Illumina raw reads were trimmed using Trim Galore version 0.6.7 (https://github.com/FelixKrueger/TrimGalore) with default parameters. *De novo* assembly of Illumina-only read sets was performed using Shovill version 1.1.0 (https://github.com/tseemann/shovill). Long reads were filtered with Filtlong version 0.2.1 using default parameters (https://github.com/rrwick/Filtlong). Hybrid assemblies, combining short and long reads, were performed using Unicycler version 0.4.8 (20). Genome assembly quality was evaluated using QUAST version 5.0.2 (21). Annotation of the *de novo* short-read and hybrid genome assemblies was conducted using Bakta version 1.9.3 (22). Assembly metrics, isolates metadata, and accession numbers are provided in Tables S1 and S2.

### *In silico* taxonomic identification, MLST, resistance genes, and plasmids

Taxonomic identification was performed using GTDB-Tk version 2.3.2 (23). *De novo* assemblies for all *Enterobacter cloacae* complex isolates were compared to representative publicly available complete *E. cloacae* complex genomes using MASH version 2.3 (24) (Table S3). The top hit based on matching hashes was used to determine the species of each isolate. Multi-locus sequence typing (MLST) was conducted using the mlst tool (https://github.com/tseemann/mlst) against PubMLST typing schemes for *Klebsiella pneumoniae*, *E. cloacae*, and *Citrobacter freundii*. This provided sequence type (ST) assignments. Antimicrobial resistance genes were identified using AMRFinder version 3.12.8 (25), with the database version 2024-01-31.1. Plasmid replicons were detected using the PlasmidFinder database version 2023-01-18 (26) using a minimum coverage of 90% and minimum nucleotide identity of 90%.

### Phylogenetic analysis

To evaluate the phylogenetic relationships between isolates and assess their potential clonal relationships, we used Snippy version 4.6.0 (https://github.com/tseemann/snippy) to align trimmed Illumina reads against a single internal reference genome for each species. The internal reference chosen was the isolate with the earliest date of isolation (BB1600 for *E. hormaechei* and BB1100 for *K. pneumoniae*). For *Mixta calida*, obtained from environmental sampling, the hybrid assembly with the best assembly metrics (BB1623) was used as a reference. We used Gubbins version 3.2.1 (27) to identify and exclude regions of recombination. The total number of core single nucleotide polymorphisms (core SNPs) in the filtered whole-genome sequence alignment was extracted using SNP-sites version 2.5.1 (28). This alignment was used as input for IQ-TREE version 2.2.6 (29) to construct maximum-likelihood phylogenetic trees, with a general time-reversible model and applying 1,000 ultra-fast bootstrap replicates (-B 1,000). SNP distances were determined using SNP-dists (https://github.com/tseemann/snp-dists). Phylogenetic trees were visualized and annotated with metadata columns using the R package ggtree version 3.4.0 (30) and edited with Inkscape version 1.2.1 (https://inkscape.org/). We used the number of SNPs between isolates to create minimum spanning trees using GrapeTree version 1.5.0 (31). To provide a clinical context, we included all Spanish *E. hormaechei* ST171 (*n* = 6) and *K. pneumoniae* ST11 (*n* = 514) genomes available in Pathogenwatch (accessed 15 July 2025; Table S4) and processed them using the same pipeline described above.

### IncR plasmid comparisons and analysis

Complete IncR plasmids were obtained from the hybrid assemblies. These plasmids were circularized using the IncR plasmid replicon (DQ449578) with Circlator version 1.5.5 (32) and annotated using Bakta version 1.9.3 (22). Using the earliest isolated plasmid pBB1600 as the reference, all identified complete and circularized IncR plasmids, as well as draft assemblies from isolates with only Illumina reads, were aligned using BLASTn version 2.16.0 and the Proksee tool (https://proksee.ca/). The average nucleotide identity between plasmids was calculated using FastANI version 1.33 (33). To predict plasmid mobility, we used MOB-typer from MOB-suite version 3.1.8 (34, 35). Briefly, MOB-typer predicts mobility based on annotations of relaxase (mob), mating pair formation (MPF) complex, and *oriT* genes. Plasmids were categorized as conjugative (containing both mob and MPF), mobilizable (containing either mob or *oriT* but no MPF), and non-mobilizable (lacking both relaxase and *oriT*). For a comprehensive analysis, we retrieved all plasmids containing the “IncR” replicon from the PLSDB database (2023_11_03_v2) (36) with associated metadata. We computed pairwise MASH version 2.3 (24) between the reference IncR plasmid pBB1600 and all IncR plasmids in PLSDB. The resulting distribution showed that distances ≤ 0.02 occurred infrequently and formed a distinct left tail, while counts increased steeply above this value (Fig. S1). We used this empirical elbow as a conservative threshold to select related plasmids for downstream analysis. The retrieved IncR plasmids (Table S5) were annotated with Bakta version 1.9.3 (22). A comparative analysis was performed using the web version of Clinker (https://cagecat.bioinformatics.nl/tools/clinker), allowing for visualization of gene arrangements and comparative genomics.

### Conjugation assay

Conjugation assays were performed using three IncR plasmid-carrying isolates (*K. pneumoniae* BB1100, *E. hormaechei* subsp. *xiangfangensis* BB1600, and *E. coli* DH5α BB1104) as donor strains and two laboratory strains (*E. coli* K-12 MG1655 and *Klebsiella quasipneumoniae* ATCC700603) as recipients. The recipient strains were transformed with a modified pUC19 vector (GenBank accession M77789.2) in which the ampicillin resistance marker was replaced with a zeocin resistance marker, enabling the selection of both recipients and transconjugants. In addition to the IncR plasmids, isolate BB1100 harbored a conjugative IncFII(pKP91)/IncFIB(K) plasmid, while isolate BB1600 carried both an IncC plasmid and a conjugative IncHI1A(NDM-CIT)/IncHI1B(pNDM-CIT) plasmid. *E. coli* DH5α BB1104 contained no additional plasmids.

Donor and recipient strains were cultured overnight at 37°C with agitation from single colonies, using gentamicin sulfate (50 mg/L, Sigma) for the donors and zeocin (200 mg/L, InVivoGen) for the recipients. Cultures were mixed in equal proportions based on their OD_600_, centrifuged for 8 minutes at 6,000 rpm, and then resuspended in either 10 µL of LB for solid medium assays or 500 µL of LB (without agar) for liquid medium assays. The mixtures were aliquoted into 24-well plates in triplicate, with 1 mL of LB agar added for solid conjugation experiments (37). Conjugation pairs were co-cultured for 18 hours at 37°C without agitation. Following incubation, the mixtures were resuspended in 500 µL of saline solution, and serial 10-fold dilutions were plated on selective media. Conjugation rates were determined as the number of transconjugants (quantified on plates supplemented with 200 mg/L zeocin and 50 mg/L gentamicin) per donor (quantified on plates supplemented with 50 mg/L gentamicin).

## RESULTS

### *armA*-positive *Enterobacter hormaechei* subsp. *xiangfangensis* ST171 carrying a conserved IncR plasmid caused equine surgical-site infections between 2011 and 2020

From 5,149 clinical samples processed at the veterinary teaching hospital between 2011 and 2020, we recovered 789 Enterobacterales isolates. Sixty-eight (8.6%) met the phenotypic inclusion criteria for resistance to aminoglycosides (see Materials and Methods), and four of those grew on medium supplemented with high concentrations of aminoglycosides. All four came from post-surgical wound infections in horses and belonged to the *Enterobacter cloacae* complex. We designated the isolates BB1600 (2015) and BB1601-BB1603 (all 2019) (Table S1). The four *E. cloacae* complex isolates exhibited resistance to multiple antibiotics, including ampicillin, azithromycin, chloramphenicol, ciprofloxacin, nalidixic acid, sulfamethoxazole, tetracycline, and trimethoprim (Table 1). They also showed high-level resistance to the 4,6-disubstituted 2-deoxystreptamine aminoglycosides gentamicin, amikacin, and kanamycin (Table S6). PCR screening confirmed the presence of the *armA* gene in all four isolates.

**TABLE 1:** MICs for the *E. hormaechei* subsp. *xiangfangensis* ST171 isolates

ID	MIC (mg/L)^*a*^												
	AMP	AZM	CAZ	CHL	CIP	CST	CTX	MEM	NAL	SMX	TET	TGC	TMP
BB1600	**>64**	**64**	2	**32**	**8**	<1	1	0.12	**>128**	**>1,024**	**>64**	0.5	**>32**
BB1601	**>64**	**>64**	2	**64**	**>8**	<1	1	0.12	**>128**	**>1,024**	**>64**	2	**>32**
BB1602	**>64**	**>64**	2	**64**	**>8**	<1	1	0.12	**>128**	**>1,024**	**>64**	2	**>32**
BB1603	**>64**	**64**	2	**32**	**8**	<1	1	0.06	**>128**	**>1,024**	**>64**	1	**>32**

^*a*^ AMP, ampicillin; AZM, azithromycin; CAZ, ceftazidime; CHL, chloramphenicol; CIP, ciprofloxacin; CST, colistin; CTX, cefotaxime; MEM, meropenem; NAL, nalidixic acid; SMX, sulfamethoxazole; TET, tetracycline; TGC, tigecycline; TMP, trimethoprim. Values in bold indicate resistance according to EUCAST or CLSI guidelines.

**Fig 1:**
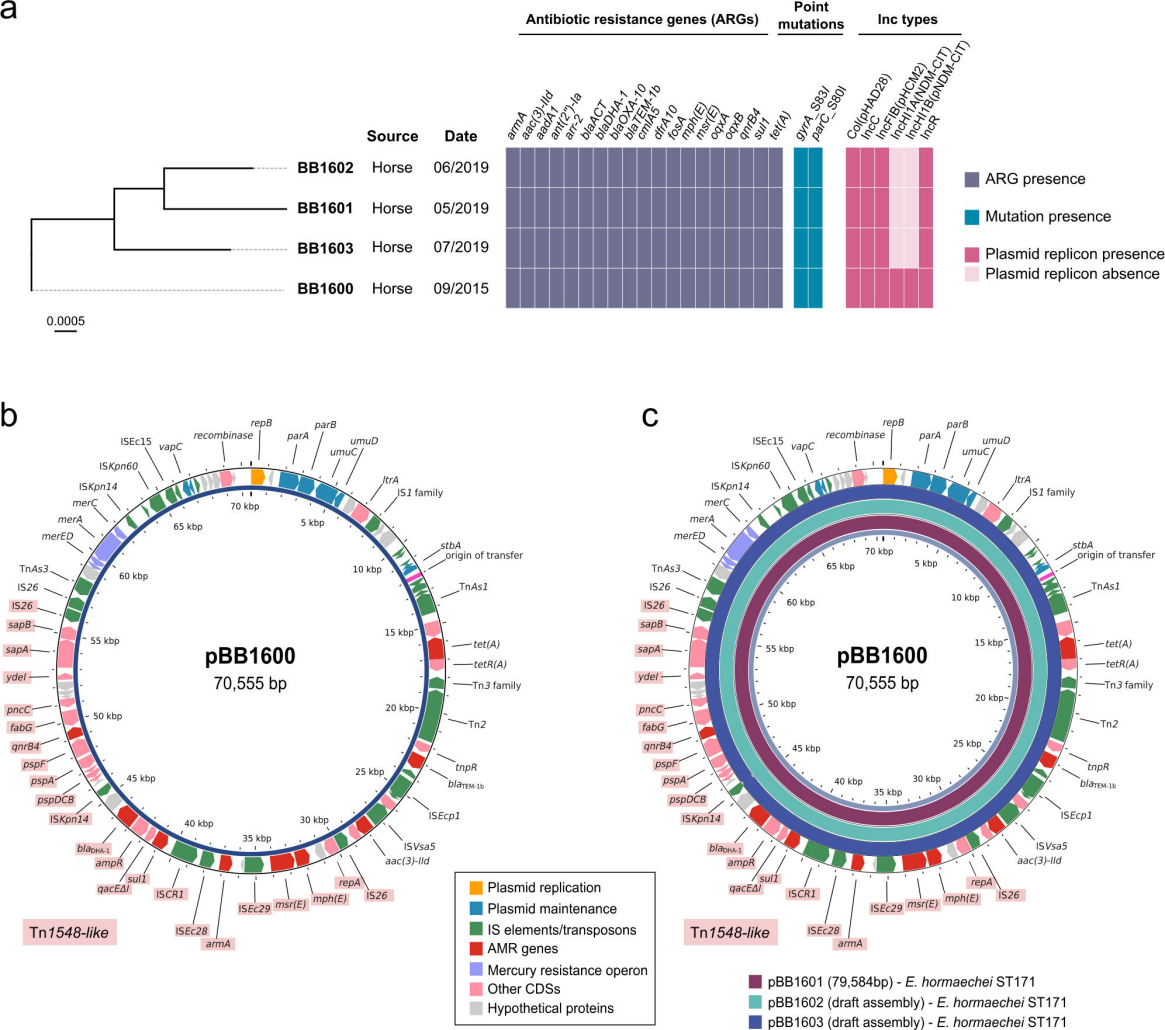
(**a**) Phylogenetic tree of four *E. hormaechei* ST171 isolates. The source and isolation dates are shown in columns. The heat maps display the presence (solid color) or absence (clear color) of antimicrobial resistance genes, point mutations, and plasmid replicon (Inc) types. (**b**) Genetic map of pBB1600 IncR plasmid. Genes are denoted as arrows. Plasmid replication genes are depicted in orange, plasmid maintenance genes in blue, IS elements and transposons in green, ARGs in red, the *mer* operon in purple, and other coding sequences (CDSs) in pink. Hypothetical proteins are shown in gray. The Tn*1548*-like region is shaded in red. (**c**) Circular BLAST alignment of the complete *E. hormaechei* IncR plasmid (pBB1601) and *E. hormaechei* draft assemblies (BB1602 and BB1603) using pBB1600 as reference.

WGS analysis identified these isolates as *E. hormaechei* subsp. *xiangfangensis*, all belonging to ST171. The WGS data revealed identical resistance gene profiles and highly similar plasmid content among the isolates, with BB1600 carrying additional IncHI1A and IncHI1B replicons (Fig. 1a). The SNP analysis showed 36–52 SNP differences among the *E. hormaechei* ST171 isolates. Long-read sequencing of BB1600 and BB1601 enabled the identification of the *armA* gene on an approximately 70 kb IncR plasmid (Fig. 1b). This plasmid contained *armA* within a Tn*1548*-like structure of approximately 27 kb flanked by IS*26*, along with other resistance genes such as *msr(E*), *mph(E*), *sul1*, *bla*_DHA-1_, and *qnrB4*. Additional resistance genes found on this plasmid included *tet(A*), *bla*_TEM-1b_, and *aac(3)-IId*. Comparative analysis with the short-read assemblies of BB1602 and BB1603 confirmed that all isolates harbored the same IncR plasmid, with a nucleotide identity ranging from 99.2% to 99.8%, when compared to the reference plasmid pBB1600 (Fig. 1c).

### IncR plasmids in *E. hormaechei* ST171 are linked to a previous *K. pneumoniae* ST11 outbreak

**Fig 2:**
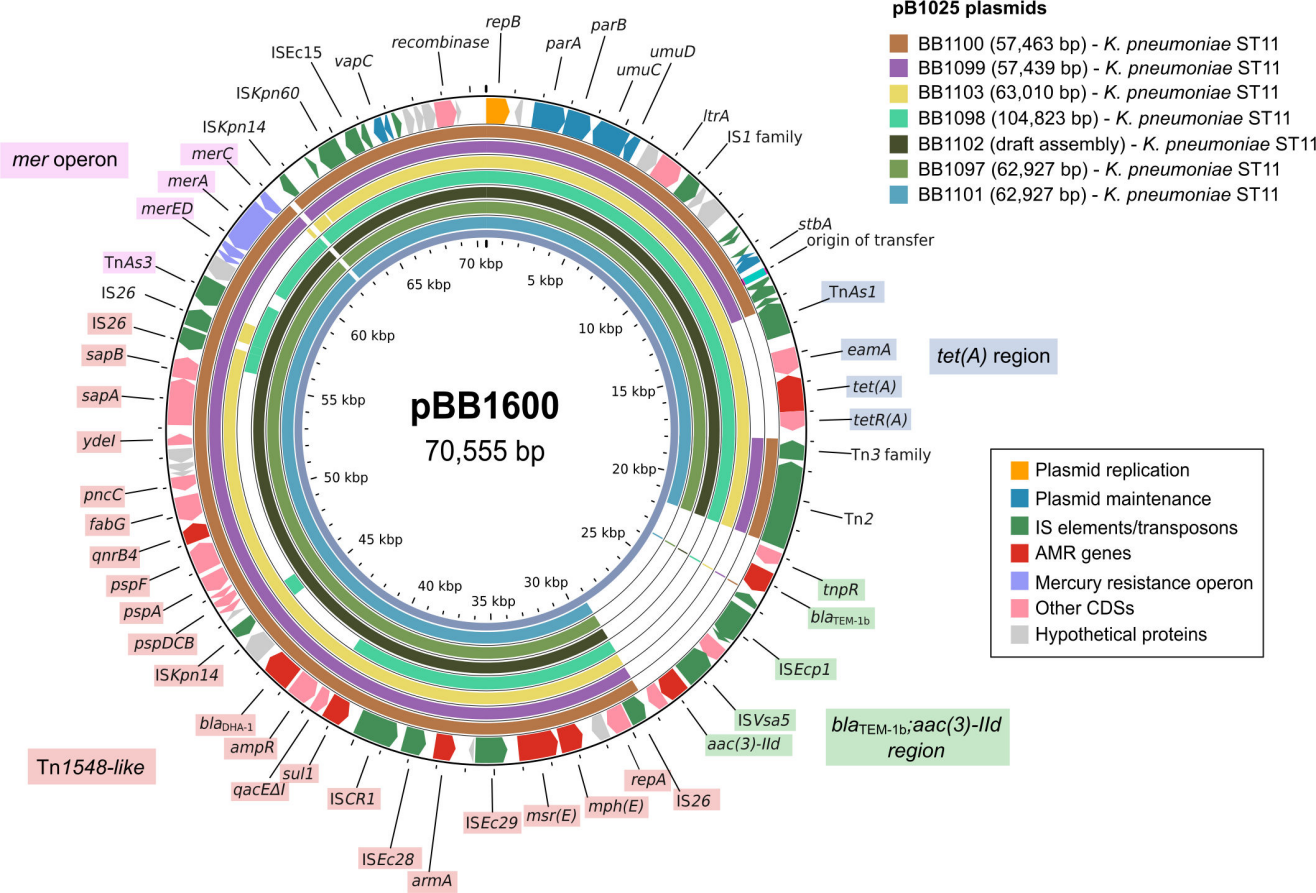
Circular BLAST alignment of the complete *K. pneumoniae* IncR pB1025 plasmids (BB1100, BB1099, BB1103, BB1098, BB1097, and BB1101) and draft assemblies (BB1102) using pBB1600 as reference. The outer ring represents pBB1600, while the inner rings correspond to the different *K. pneumoniae* IncR pB1025 plasmids. The Tn*1548*-like region is shaded in red, the *tet(A*) region in blue, the *bla*_TEM-1b_ and *aac(3)-IId* region in green, and the *mer* operon in pink.

Between 2008 and 2010, an outbreak of highly aminoglycoside-resistant *K. pneumoniae* ST11 was reported in dogs and cats (*n* = 7) at the same Veterinary Teaching Hospital. These isolates carried the *armA*, *bla*_DHA-1_, and *qnrB4* genes on an IncR plasmid (designated as pB1025), marking the first description of the *armA* gene in companion animals (38). At that time, the presence of these ARGs and plasmids was confirmed by PCR. In this study, we sequenced the *K. pneumoniae* ST11 isolates using both short and long-read sequencing technologies. Phylogenetic analysis revealed that these isolates were related, exhibiting 13–44 SNP differences and similar resistance gene and plasmid profiles (Fig. S2). All isolates carried an IncR plasmid highly similar to the IncR plasmid identified in *E. hormaechei* ST171, with nucleotide identities ranging between 96.3% and 99.7% (Fig. 2).

The IncR plasmids identified in *K. pneumoniae* ST11 were generally smaller in size (57.4–63 kb) compared to those found in *E. hormaechei* ST171, except for pB1025_BB1098, which was 104.8 kb because it had captured a 60 kb fragment of a co-resident IncFII(pKP91)/IncFIB(K) plasmid (Fig. S3). This region likely resulted from recombination events facilitated by common elements shared by both plasmids, such as the *sul1* gene and IS*Kpn14*. Comparative analysis showed that the Tn*1548*-like element containing the *armA* gene in the *K. pneumoniae* ST11 plasmids was identical to that found in *E. hormaechei* ST171. However, other regions of the plasmid structure exhibited variations. pB1025_BB1100 and pB1025_BB1099 lacked a 5 kb region containing Tn*As1* and the tetracycline resistance gene *tet(A*), which appeared in the other plasmids. Additionally, none of the *K. pneumoniae* ST11 plasmids contained a 7 kb region carrying the *bla*_TEM-1b_ and *aac(3)-IId* genes, which were present in the *E. hormaechei* IncR plasmids. pB1025_BB1103 also lacked the *mer* operon region (Fig. 2). Despite these structural variations, which highlight the adaptive flexibility of this plasmid, the close genetic relationship between the IncR plasmids found in *E. hormaechei* ST171 and those from the *K. pneumoniae* ST11 outbreak suggested that this plasmid had been able to mobilize and maintain itself within the veterinary hospital environment over several years.

Comparison with Spanish human genomes showed no close link for *E. hormaechei* ST171 (>17,000 SNPs), but the veterinary *K. pneumoniae* ST11 isolates clustered within the national ST11 population (median 61 SNPs, range 41–693; Fig. S4b). IncR replicons were present in 81% (418/514) of human ST11 genomes, with six carrying the same ARG combination (*armA*, *bla*_DHA-1_, and *qnrB4*) found in the veterinary isolates.

### IncR plasmids can use the conjugative machinery of cohabiting plasmids to mobilize

Despite the hypothesis of possible horizontal transfer of the IncR plasmid from *K. pneumoniae* to *E. hormaechei*, our analysis revealed that the IncR plasmids identified in this study lacked the necessary conjugative machinery (Fig. 1b). Using the mob-typer tool, we assessed the mobility potential of these plasmids and categorized all IncR plasmids as mobilizable, presenting an origin of transfer (*oriT*). To explore the potential for conjugative transfer, we examined the plasmids cohabiting with IncR plasmids in both species. In *K. pneumoniae* isolates, all carried an additional multireplicon plasmid, IncFII(pKP91)/IncFIB(K), which was categorized as conjugative. In *E. hormaechei* isolates, several plasmids were present in addition to IncR. The two isolates sequenced with Nanopore technology (BB1600 and BB1601) contained a conjugative IncC plasmid, and BB1600 additionally presented an IncHI1A(NDM-CIT)/IncHI1B(pNDM-CIT) plasmid, also categorized as conjugative. Thus, the multidrug-resistant *K. pneumoniae* ST11 and *E. hormaechei* ST171 isolates in our collection could mobilize their *armA*-carrying IncR plasmid via co-resident conjugative IncF, IncC, or IncHI1 plasmids.

To experimentally validate the hypothesis that IncR plasmids can exploit the conjugative machinery of cohabiting plasmids, we conducted conjugation experiments using three donor strains: *K. pneumoniae* BB1100 [carrying both the IncR plasmid and the conjugative IncFII(pKP91)/IncFIB(K) plasmid], *E. hormaechei* subsp. *xiangfangensis* BB1600 [harboring the IncR plasmid along with the conjugative IncC and IncHI1A(NDM-CIT)/IncHI1B(pNDM-CIT) plasmids], and *E. coli* DH5α BB1104 (which contained only the IncR plasmid). In the solid medium, the IncR plasmid was successfully transferred from the donor strain *E. hormaechei* BB1600 to both recipient strains; *E. coli* MG1655 exhibited a conjugation rate of 7.2 × 10^−8^, while *K. quasipneumoniae* ATCC700603 showed a rate of 5.24 × 10^−8^. However, no transfer of the IncR plasmid was detected from *K. pneumoniae* BB1100 to either recipient despite its co-carriage of a conjugative plasmid. Likewise, *E. coli* DH5α BB1104, lacking any conjugative plasmids, did not mediate mobilization of the IncR plasmid.

### Environmental sampling of hospital surfaces reveals multispecies and polyclonal maintenance of *armA* via the IncR plasmid

**Fig 3:**
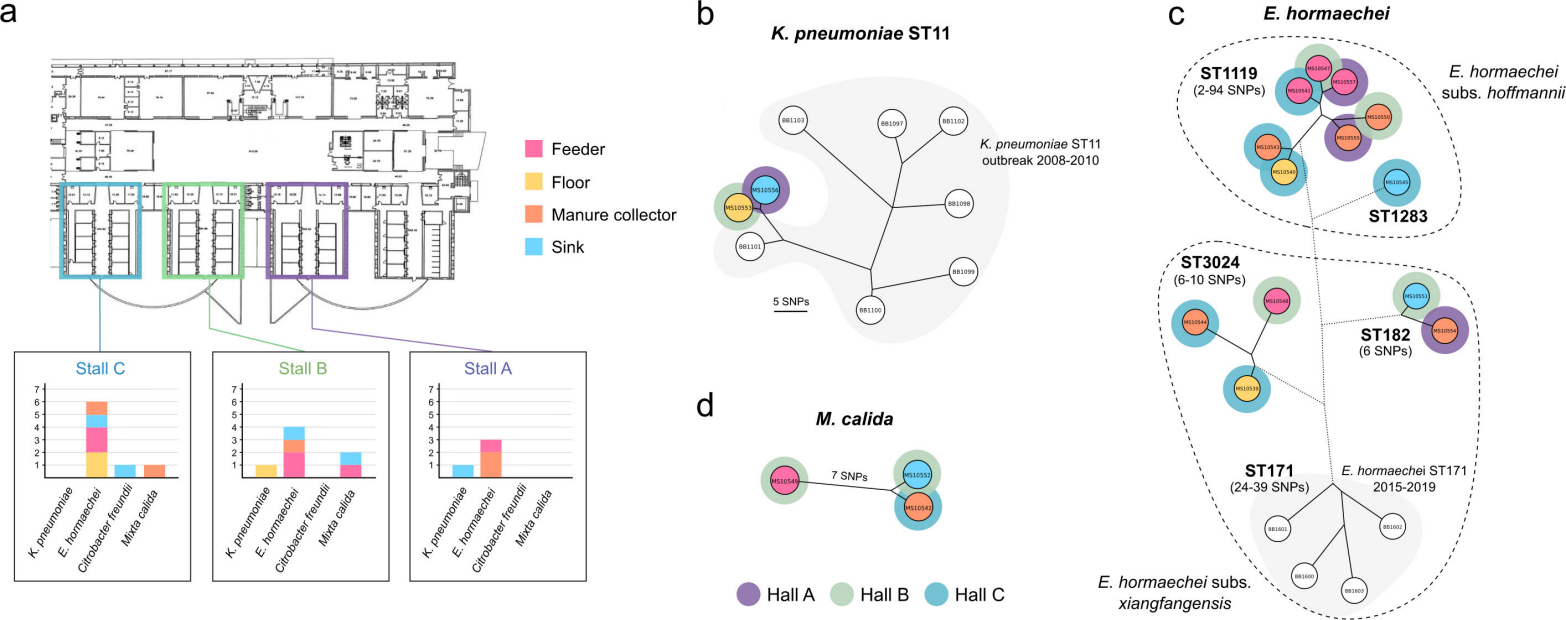
(**a**) Representation of the three horse stalls sampled at the veterinary hospital (A in purple, B in green, and C in blue). In each stall, a bar plot shows the distribution of isolates of each bacterial species, colored according to the surface from which they were isolated. Panels **b**, **c**, and **d** show minimum spanning trees constructed from SNP distances when aligning isolates against an internal reference (BB1100 for *K. pneumoniae*, BB1600 for *E. hormaechei*, and BB1623 for *M. calida*). Strains isolated prior to environmental sampling are included in the MSTs and are shaded in gray. Isolates from environmental sampling are colored according to the surface where they were isolated and surrounded by a colored circle corresponding to the stall (A, B, or C).

In 2022, 12 surface samples were collected from three horse stalls (designated as A, B, and C) in the veterinary hospital, targeting sinks, floors, feeders, and manure collectors. Bacteria resistant to high levels of aminoglycosides and carrying the *armA* gene were detected in nearly all locations (Table S7). Interestingly, the environmental sampling revealed a broad diversity of bacterial species across different surfaces and stalls (Fig. 3a). The predominant species found was *E. hormaechei* (specifically *E. hormaechei* subsp. *hoffmannii* and *E. hormaechei* subsp. *xiangfangensis*) in all stalls (A: *n* = 3; B: *n* = 4; and C: *n* = 6) and across all surfaces. Additionally, *K. pneumoniae* (*n* = 2) was identified in stalls A and B (sink and floor, respectively), *Mixta calida* (formerly *Pantoea calida*) in stalls B (*n* = 2) and C (*n* = 1), and one *Citrobacter freundii* isolate in the sink of stall C.

Nineteen isolates were sequenced using Illumina (Table S1). Short-read sequencing data were used to analyze the SNP distances among the isolates (Fig. 3b). This analysis included previously detected *E. hormaechei* ST171 isolates from horses (2015 and 2019) and the *K. pneumoniae* ST11 outbreak isolates (2008–2010). The two *K. pneumoniae* isolates from the environmental samples corresponded to ST11 and were closely related to the 2009 isolate BB1101 (9–10 SNPs). The *E. hormaechei* isolates from the environmental samples belonged to different STs: ST1119 (*n* = 7), ST3024 (*n* = 3), ST1283 (*n* = 1), and ST182 (*n* = 2) and were not related to the *E. hormaechei* ST171 identified in horses. The distribution of these clusters was not surface or stall-specific, suggesting potential cross-contamination across the hospital environment. The *M. calida* isolates from different stalls were closely related (two SNPs), and the unique *C. freundii* isolate belonged to ST8.

**Fig 4:**
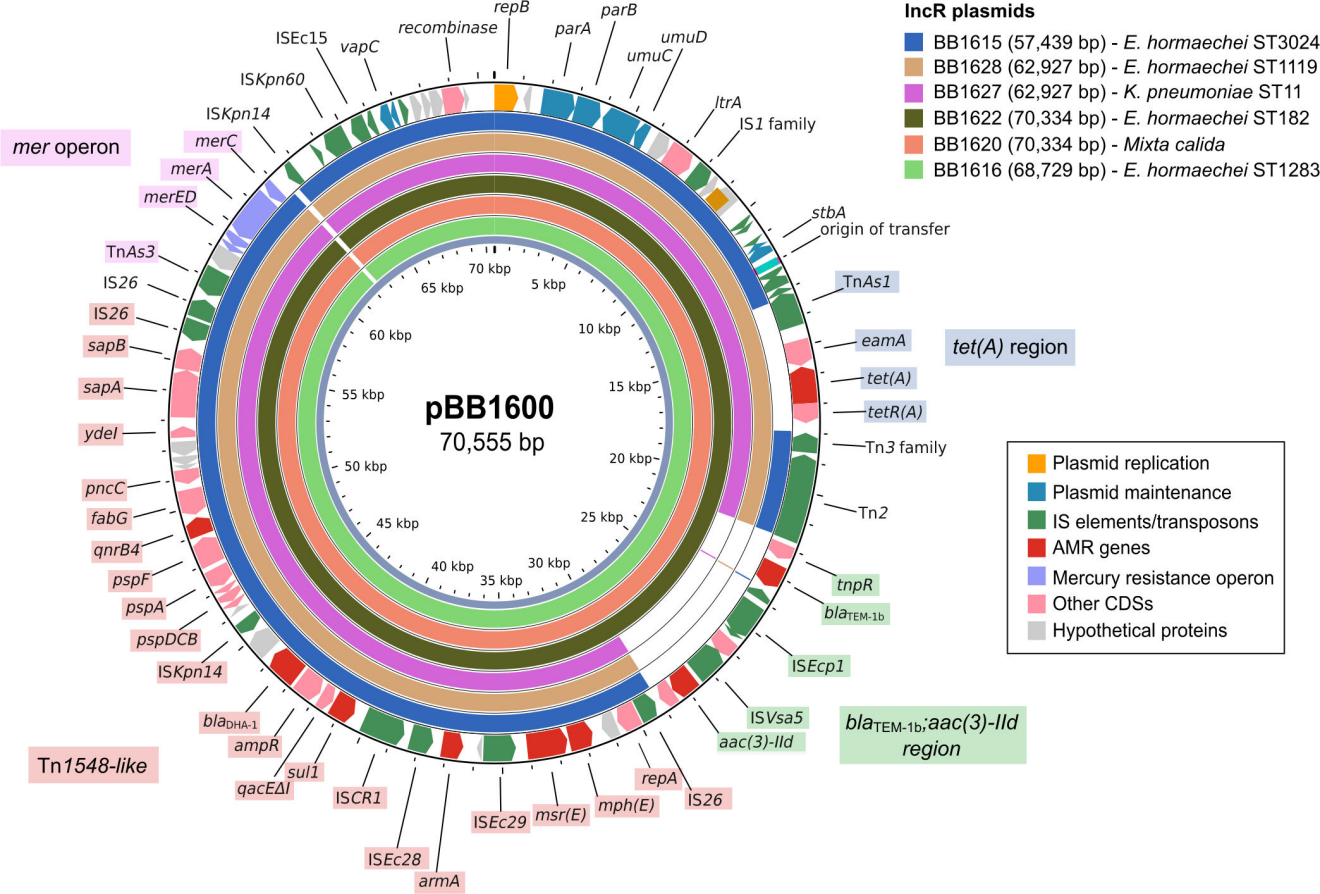
Circular BLAST alignment of the complete IncR plasmid identified in different species from the environmental sampling (*E. hormaechei*, *K. pneumoniae,* and *M. calida*). The outer ring represents pBB1600, while the inner rings correspond to the different IncR plasmids. The Tn*1548*-like region is shaded in red, the *tet(A*) region in blue, the *bla*_TEM-1b_ and *aac(3)-IId* region in green, and the *mer* operon in pink.

Out of the 19 environmental isolates, 16 (84.2%) carried an IncR plasmid. Nanopore sequencing of eight representative isolates (selecting different bacterial species and STs) confirmed that the *armA* gene was located on the IncR plasmid in most of the cases. The three exceptions were *C. freundii* ST8 (BB1617) and *M. calida* (BB1613 and BB1623). In these cases, *armA* was located on different plasmids: IncFIB(pB171)/IncFII(Yp) for *C. freundii* and IncHI2A/IncHI2/pKPC(CAV1321) for *M. calida* isolates (Fig. S5 and S6). In the remaining isolates, the IncR plasmids carrying *armA* were almost identical to the reference *E. hormaechei* ST171 plasmid pBB1600, with 99.6%–99.8% identity. Consistent with findings from the *K. pneumoniae* ST11 outbreak, some IncR plasmids lacked the *tet(A*)-containing region, as well as the *bla*_TEM-1b_ and *aac(3)-IId*-containing regions. However, plasmids nearly identical to those found in *E. hormaechei* ST171 clinical isolates from horses were identified (Fig. 4). In all cases, the Tn*1548*-like transposon containing the *armA* gene was identical and inserted at the same site. Interestingly, the two *M. calida* isolates without an IncR plasmid contained the Tn*1548*-like element on the IncHI2A/IncHI2/pKPC(CAV1321) plasmid, along with regions associated with *tet(A*), *bla*_TEM-1b_, and *aac(3)-IId* (Fig. S6).

### IncR plasmids from diverse global sources exhibit similar characteristics to those from our veterinary hospital isolates

**Fig 5:**
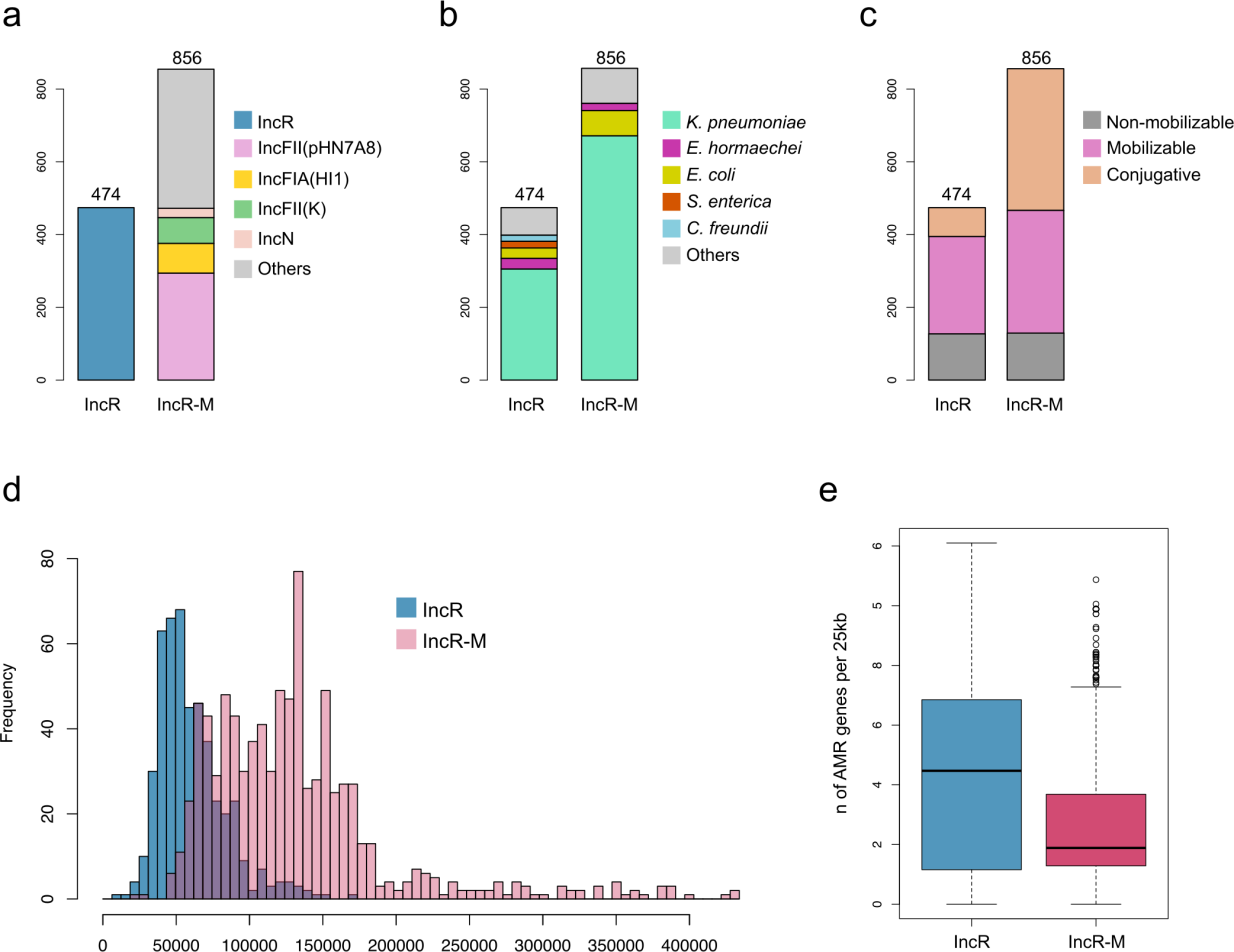
Panel **a** shows the count of IncR and multireplicon plasmids (IncR-M) in the PLSDB data set (1,330 plasmids). Panels **b** and **c** are bar plots indicating the bacterial species and predicted mobility, respectively, across the IncR and IncR-M plasmids. Panel **d** shows a histogram with the distribution of plasmid lengths in the data set. Panel **e** shows a boxplot with the number of ARGs per 25 kb across IncR and IncR-M plasmids.

To study the characteristics of the IncR plasmids found in the veterinary hospital, we analyzed 1,330 plasmids with the IncR replicon downloaded from the PLSDB database. Of these, 474 (35.6%) had a single IncR replicon, while 856 (64.4%) had multiple replicons (IncR-M), predominantly IncF replicons [IncFII(pHN7A8), IncFIA(HI1), and IncFII(K)] (Fig. 5a). The primary host species for these plasmids was *K. pneumoniae* (309; 65.1% for single IncR replicon and 677; 79% for IncR-M), with *E. hormaechei* also present but in smaller proportions (30; 6.3% for single IncR replicon and 21; 2.4% for IncR-M) (Fig. 5b). The plasmid mobility analysis revealed that more than half of the single IncR plasmids were mobilizable (268; 56.4%) and only a few were conjugative (78; 16.4%). In contrast, a significant proportion of IncR-M plasmids were conjugative (388; 45.3%) (Fig. 5c). This was correlated with their larger size, with multireplicon plasmids having a median size of 123,516 bp (interquartile range [IQR]: 87,987–153,421 bp), compared to single IncR plasmids with a median size of 55,034 bp (IQR: 44,589–71,664 bp) (Fig. 5d). IncR plasmids exhibited a higher density of resistance genes, with an average of 2.1 AMR genes per 25 kb (standard deviation [SD] 1.53), compared to IncR-M plasmids with an average of 1.3 genes per 25 kb (SD 1.08) (Fig. 5e). Despite this, the frequency of the *armA* gene was low in the data set, present in only 5 (1%) of the single IncR plasmids and 36 (4%) of the IncR-M plasmids.

**Fig 6:**
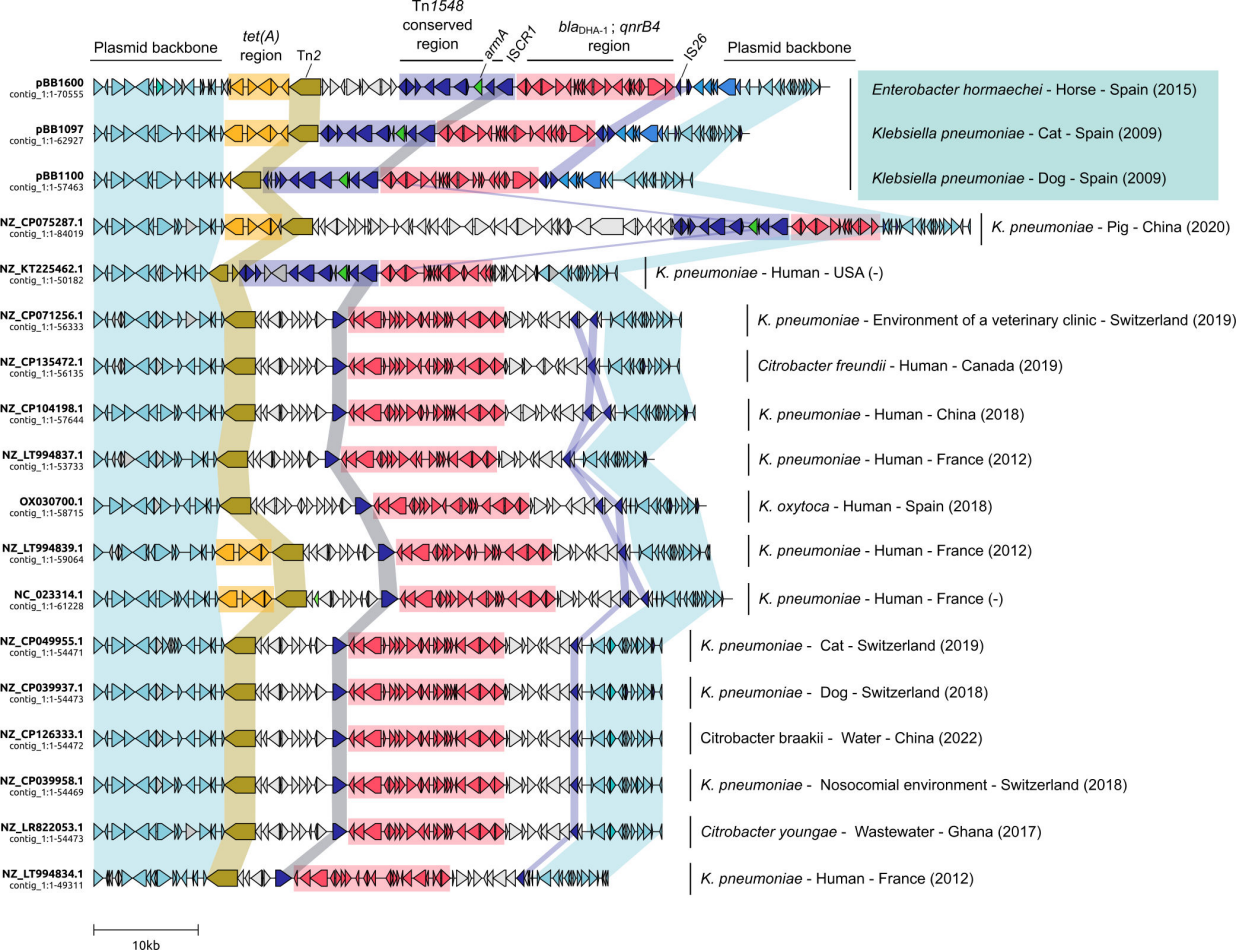
Comparison of IncR plasmids identified in this study (pBB1600, pBB1097, and pBB1100) with related IncR plasmids from the PLSDB database. The shaded areas correspond to the plasmid backbone (light blue), *tet(A*) region (yellow), Tn*2* element (brown), Tn1*548* conserved region (blue), and *bla*_DHA-1_;*qnrB4* region (red). The Tn*1548* conserved region includes IS*26-mph(E)-msr(E*)-IS*Ec29-armA* (green)-IS*Ec28*-IS*CR1*. Bacterial species, source, location, and year of isolation are indicated on the right side of the figure.

We compared our IncR plasmids to those most related in the data set (mash distance < 0.02) (Table S5). These plasmids, despite lacking an epidemiological link and originating from various global locations (China, USA, Switzerland, France, and Ghana) and sources (humans, pigs, pets, and environment), shared a common region containing *bla*_DHA-1_ and *qnrB4* associated with IS*CR1* (Fig. 6). This conserved region suggested that through recombination events involving elements such as IS*CR1* and IS*26*, a stable Tn*1548*-like structure capable of mobilizing multiple resistance genes has been formed and disseminated in IncR plasmids.

## DISCUSSION

In this study, we utilized WGS to investigate and characterize isolates with high-level aminoglycoside resistance in a Spanish veterinary hospital. While the application of WGS in epidemiological surveillance and nosocomial investigations is well-established due to its high discriminatory power and decreasing costs (39–41), its use in veterinary clinical settings remains limited. Here, we demonstrate the utility of integrating WGS with short- and long-read sequencing technologies to characterize the persistence of a resistance mechanism over more than a decade within a veterinary hospital environment.

High-level aminoglycoside resistance was rare in the hospital, with only four resistant *E. hormaechei* subsp. *xiangfangensis* isolates (0.5% of Enterobacterales between 2011 and 2020) detected from different horses in 2015 and 2019. Phylogenetic analysis showed that the four *E. hormaechei* ST171 isolates differed by 36–52 core SNPs, well above the ≤5 SNP threshold usually taken as evidence of recent direct transmission (42, 43). This makes horse-to-horse spread unlikely, although indirect acquisition from a persistent hospital reservoir cannot be ruled out. The gene conferring high-level resistance to aminoglycosides, *armA*, was located on an IncR plasmid along with other ARGs such as *bla*_DHA-1_ and *qnrB4* (Fig. 1b). An earlier outbreak of *K. pneumoniae* ST11 in the same hospital, affecting dogs and cats, also involved isolates containing the *armA* gene on an IncR plasmid (38). Through WGS, we determined that the *K. pneumoniae* and *E. hormaechei* IncR plasmids were highly similar (Fig. 2). This ARG combination (*armA*, *bla*_DHA-1_, and *qnrB4*) has rarely been described, so far only sporadically in the USA and China (44), while IncR plasmids carrying other ARGs such as *bla*_KPC-2_, *bla*_NDM-1_, *bla*_VIM-1_, and *qnrS1* are increasingly common in *K. pneumoniae* and other Enterobacterales (45–47). In comparison to human isolates, *E. hormaechei* ST171 from the hospital showed no close relationship with Spanish clinical isolates (>17,000 SNPs), but the *K. pneumoniae* ST11 isolates clustered within the dominant national lineage (Fig. S4b). Although most public ST11 assemblies were too fragmented to reconstruct plasmids, the frequent presence of IncR replicons in this lineage supports their broad circulation in Spanish *K. pneumoniae* ST11 (48).

Given the presence of similar IncR plasmids in different bacterial species, we hypothesized that specific bacteria or environmental niches might act as reservoirs for these plasmids or resistance genes. Environmental sampling from surfaces associated with horse hospitalization areas revealed various bacterial species harboring plasmids closely related to those identified in clinical isolates. These species included *Enterobacter hormaechei* subsp. *hoffmannii* and *E. hormaechei* subsp. *xiangfangensis,* as well as *K. pneumoniae* and *M. calida* (Fig. 4). Hospital environments are well-documented reservoirs of resistant pathogens in both human and veterinary settings (49, 50), not only involving surfaces and wastewater but also extending to hospital staff (51, 52). In our study, *E. hormaechei* subsp. *hoffmannii* (*n* = 8) and *E. hormaechei* subsp. *xiangfangensis* (*n* = 5) were frequently found on all sampled surfaces. These isolates belonged to different STs compared to those found in clinical cases in horses, despite carrying very similar IncR plasmids. This suggests a complex scenario of IncR plasmid dissemination in multiple clones, which then spread independently. *E. hormaechei* has been linked to various outbreaks in human and veterinary hospitals, often associated with environmental sources such as sinks (13, 53). This implies that *E. hormaechei* can survive well in hospital environments, becoming a significant cause of nosocomial infections worldwide (54, 55). Additionally, we identified *M. calida* on various surfaces. Recent evidence suggests that *M. calida* is frequently isolated from nosocomial environments, though its role in disease remains debated (56). The presence of these different species and clones across multiple stables and surfaces indicates that the hospital environment is being contaminated with these environmentally adapted bacteria, even though infections caused by them are infrequent.

Upon discovering this multispecies and polyclonal scenario of the IncR plasmid, we hypothesized that the hospital environment was experiencing two concurrent processes: minimal clonal expansion of *K. pneumoniae* ST11 and extensive horizontal mobilization. A decade after the initial outbreak (38), we identified two *K. pneumoniae* isolates closely related to the outbreak strain (9–10 SNPs; Fig. 3b), indicating limited clonal expansion, while the presence of IncR plasmids across diverse bacterial species suggests that horizontal transfer is the dominant mechanism. IncR plasmids are generally considered non-conjugative, as they lack the *tra* genes and relaxase required for self-transfer (38, 57–59). Our global analysis confirmed that these relatively small plasmids (median size of 55 kb) generally lack conjugative machinery but are predicted to be mobilizable (Fig. 5). Recent studies have demonstrated that plasmids with an *oriT* can be mobilized by cohabiting conjugative plasmids (60), a mechanism confirmed in our experiments by successful IncR transfer from *E. hormaechei* to *K. quasipneumoniae* and *E. coli*. Moreover, our findings align with recent work, indicating that mobilizable plasmids often carry high densities of antimicrobial resistance genes, serving as platforms for resistance dissemination (60, 61). We also observed that IncR plasmids are frequently co-integrated with other plasmids, particularly F-type plasmids, which may further facilitate their spread, a phenomenon that has been observed in previous studies (62).

The dynamic nature of the IncR plasmids, characterized by numerous and diverse resistance genes and mobile genetic elements such as ISs and transposons, suggests that these plasmids undergo frequent genetic rearrangements, as seen in our isolates (Fig. 2 and 4). Additionally, we identified similar IncR plasmids in public genomes that shared a common structure containing *bla*_DHA-1_ and *qnrB4* (Fig. 6), a combination frequently reported in *Enterobacteriaceae* worldwide (44, 63, 64) and often associated with IncH-like plasmids (65–68). This region may have originated through recombination with cohabiting IncH plasmids or via IS*CR1*-mediated rearrangements (63, 69), though further work is needed to clarify these mechanisms.

This study shows that *armA*-carrying IncR plasmids have persisted for more than a decade in a veterinary hospital, moving between *K. pneumoniae* ST11, *E. hormaechei* ST171, and multiple environmental Enterobacterales via helper conjugative plasmids. As plasmid-mediated dissemination is harder to control than clonal spread, ongoing genomic surveillance and judicious aminoglycoside use in equine medicine are vital to prevent further spread.

## Data Availability

All sequencing data generated in this study are publicly available at the National Center for Biotechnology Information (NCBI) under BioProject PRJNA1256697. Individual accession numbers are listed in Table S1.

## SUPPLEMENTAL MATERIAL

Supplemental figures jcm.00673-25-s0001.docx Figures S1 to S6.

Supplemental tables jcm.00673-25-s0002.xlsx Tables S1 to S7.

ASM does not own the copyrights to Supplemental Material that may be linked to, or accessed through, an article. The authors have granted ASM a non-exclusive, world-wide license to publish the Supplemental Material files. Please contact the corresponding author directly for reuse.
