# Supplementary material for: Persistent multispecies dissemination of armA-carrying IncR plasmids among clinical and environmental bacterial populations in a Spanish veterinary hospital
Source: J Clin Microbiol. 2025 Oct 9;63(11):e00673-25. doi: 10.1128/jcm.00673-25 (PMC12607884; doi:10.1128/jcm.00673-25)
Supplement: Supplemental figures — Figures S1 to S6. [file jcm.00673-25-s0001.docx]

**Persistent multispecies dissemination of *armA*-carrying IncR plasmids among clinical and environmental bacterial populations in a Spanish veterinary hospital**

Carlos Serna, Mario Pulido-Vadillo, Bosco R. Matamoros, Javier F. Favieres, Natalia Montero, Claudia García Berdún, Marta E. García, Jose L. Blanco, Jose F. Delgado-Blas, Bruno Gonzalez-Zorn

**Supplementary Figures**

**Figure S1**: Histogram of mash distances between the reference IncR plasmid pBB1600 and all IncR plasmids in the PLSDB database.

**Figure S2**: Phylogenetic tree of seven *K. pneumoniae* ST11 isolates from a previous outbreak in the same veterinary hospital

**Figure S3**: Genetic comparison between the plasmids carried by isolates BB1099 and BB1098.

**Figure S4**: Nationwide phylogenetic context of veterinary hospital isolates.

**Figure S5**: Genetic map of *Citrobacter freundii* pBB1617 IncFIB/IncFII plasmid.

**Figure S6**: Genetic map of *Mixta calida* pBB1623 IncHI2A/InHI2/pKPC-CAV1321 plasmid.

**
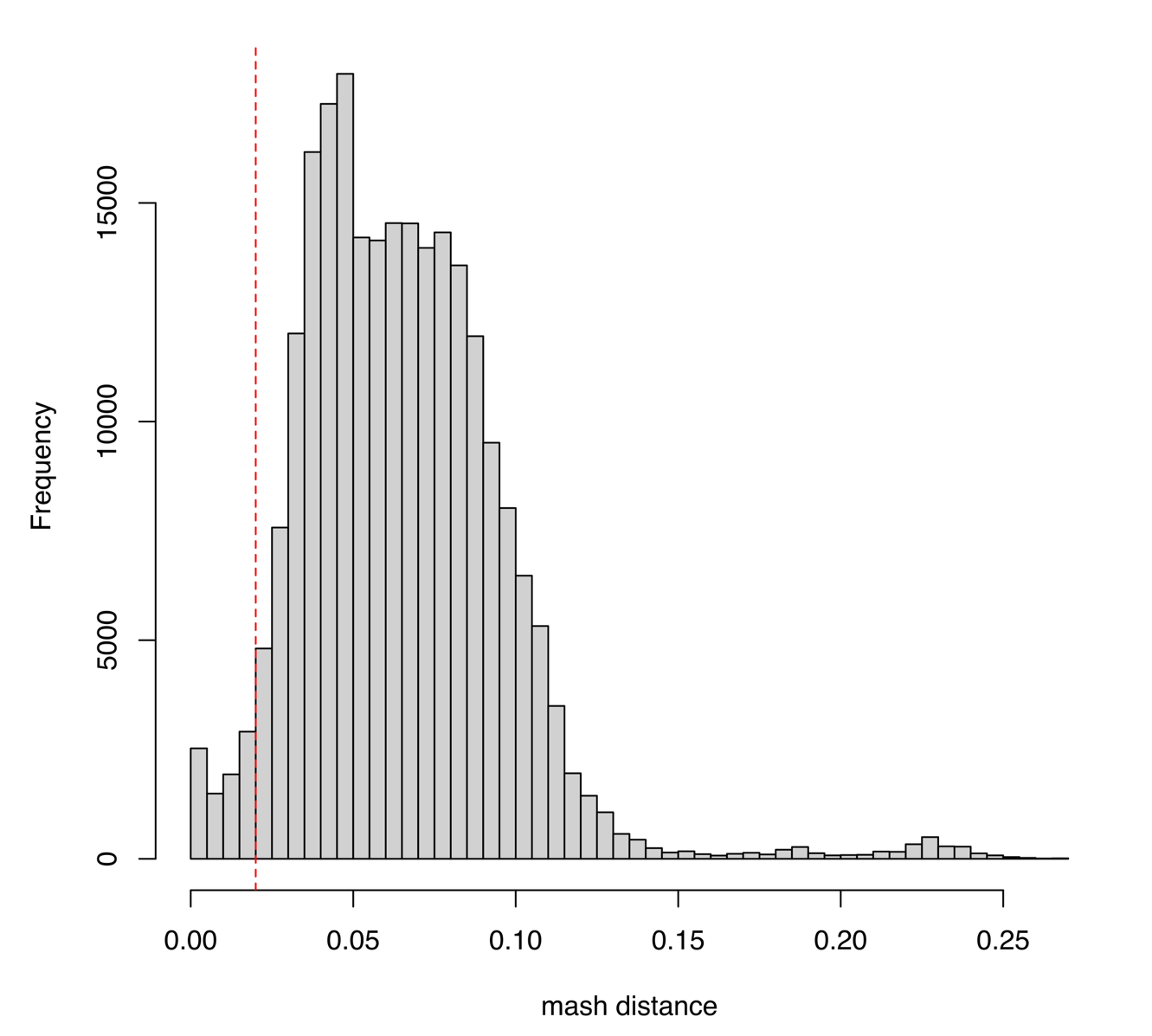
**

**Figure S1**. Histogram of mash distances between the reference IncR plasmid pBB1600 and all IncR plasmids in the PLSDB database. The red dashed line indicates the threshold (≤ 0.02) used to define closely related plasmids for comparative analysis.

**
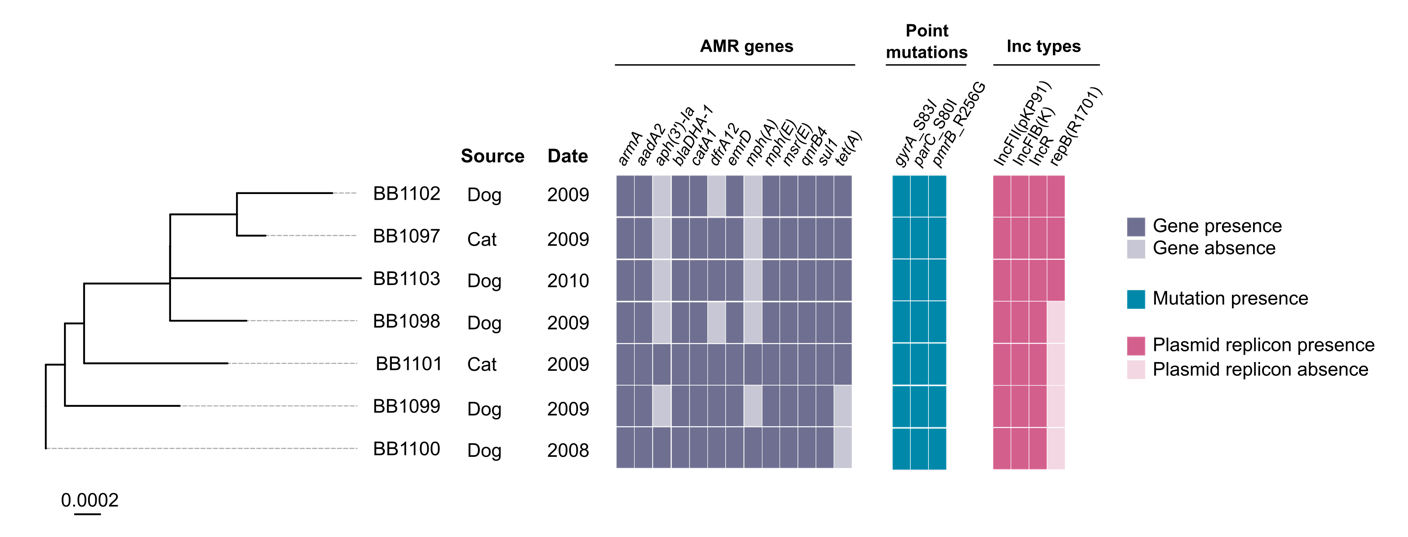
**

**Figure S2.** Phylogenetic tree of seven *K. pneumoniae* ST11 isolates from a previous outbreak in the same veterinary hospital. The source and isolation dates are shown in columns. The heat maps display the presence (solid color) or absence (clear color) of antimicrobial resistance genes (ARGs), point mutations and plasmid replicon (Inc) types.

**
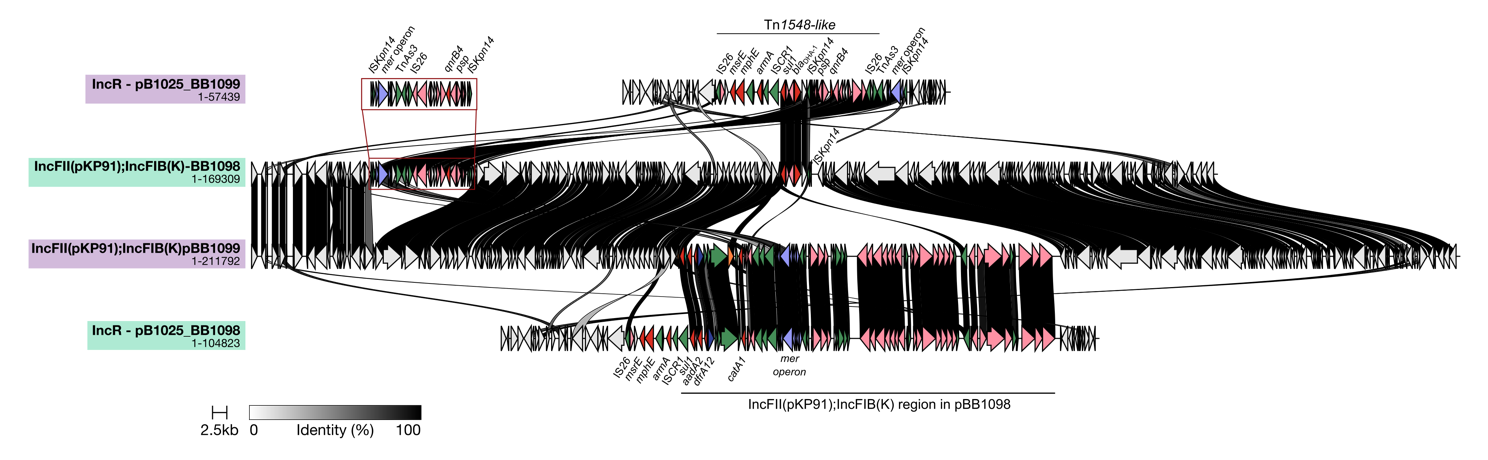
**

**Figure S3.** Genetic comparison between the plasmids carried by isolates BB1099 (purple label on the left) and BB1098 (light green label on the left). Both isolates carried an IncR plasmid and an IncFII-IncFIB(K) multireplicon plasmid. The IncR plasmid pB1025_BB1098 (bottom of the figure) incorporates a region of approximately 60 kbp found in the IncFII-IncFIB plasmid. Conversely, the plasmid cohabiting with IncR pB1025_BB1098 lacks this region but incorporates the Tn*1548*-like region of the IncR plasmid.

**
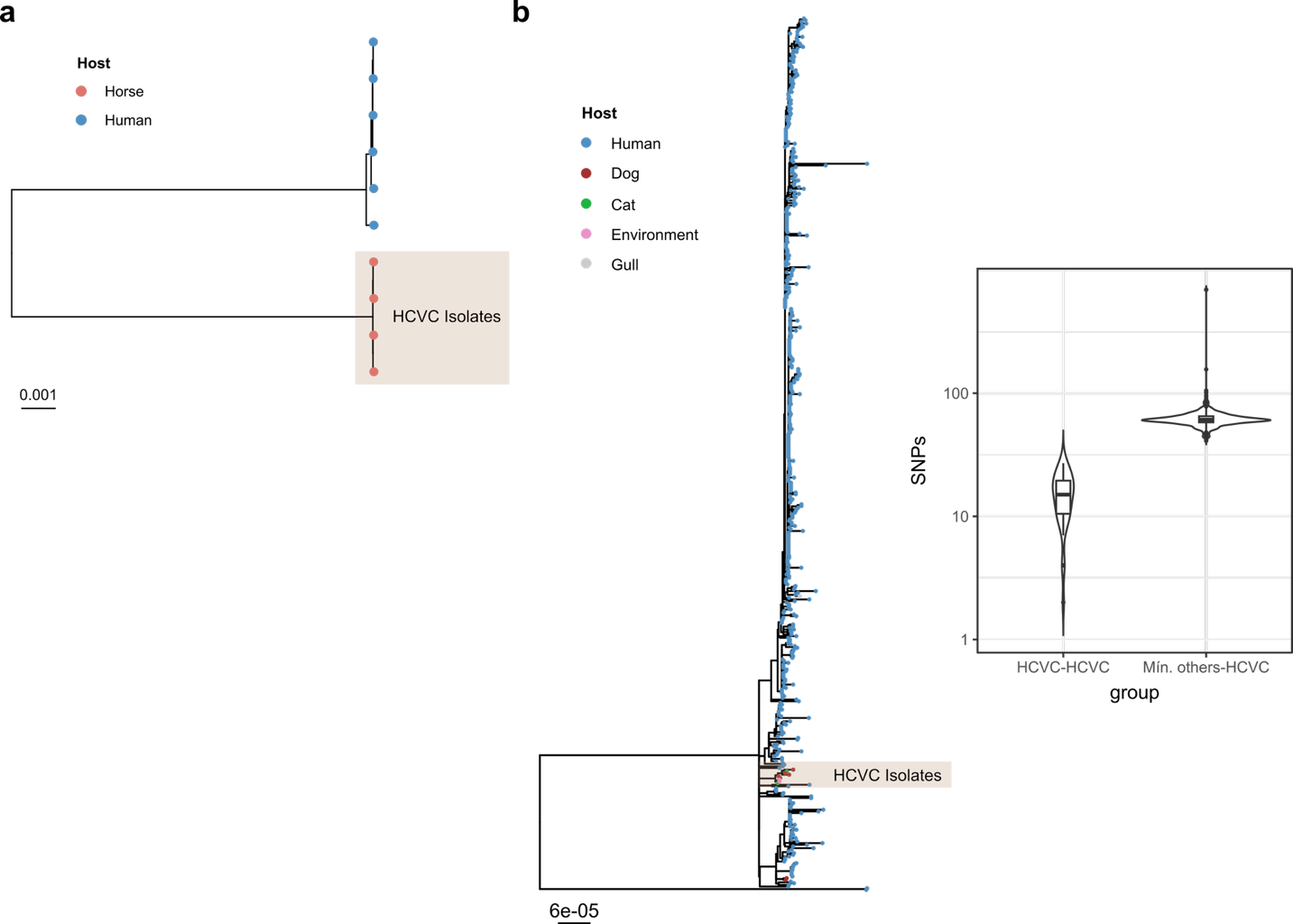
**

**Figure S4.** Nationwide phylogenetic context of veterinary hospital isolates. Core genome SNP-based analysis and maximum likelihood trees annotated with source metadata of (a) *Enterobacter hormaechei* ST171 (n=6) and (b) *Klebsiella pneumoniae* ST11 (n=514) genomes from Pathogenwatch, including veterinary hospital isolates (HCVC, highlighted in brown). The violin plot shows SNP distances between veterinary isolates (HCVC-HCVC) and between veterinary and external Spanish isolates (others-HCVC).

**
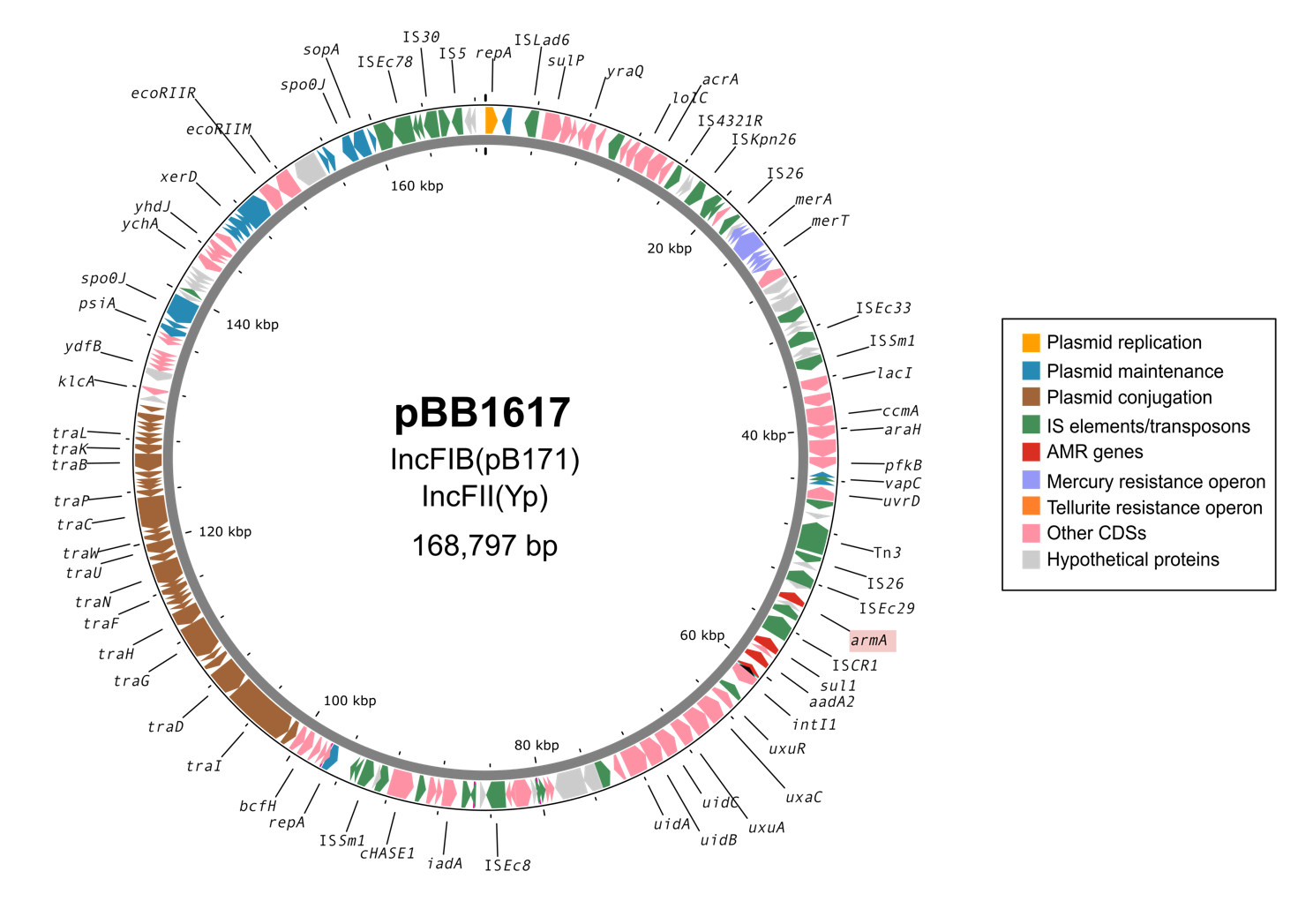
**

**Figure S5.** Genetic map of *Citrobacter freundii* pBB1617 IncFIB/IncFII plasmid. Genes are denoted as arrows. Plasmid replication genes are depicted in light orange, plasmid maintenance genes in blue, plasmid conjugation genes in brown, IS elements and transposons in green, ARGs in red, the *mer* operon in purple, tellurite resistance operon in dark orange and other coding sequences (CDSs) in pink. Hypothetical proteins are shown in grey. The *armA* gene is shaded in red.

**
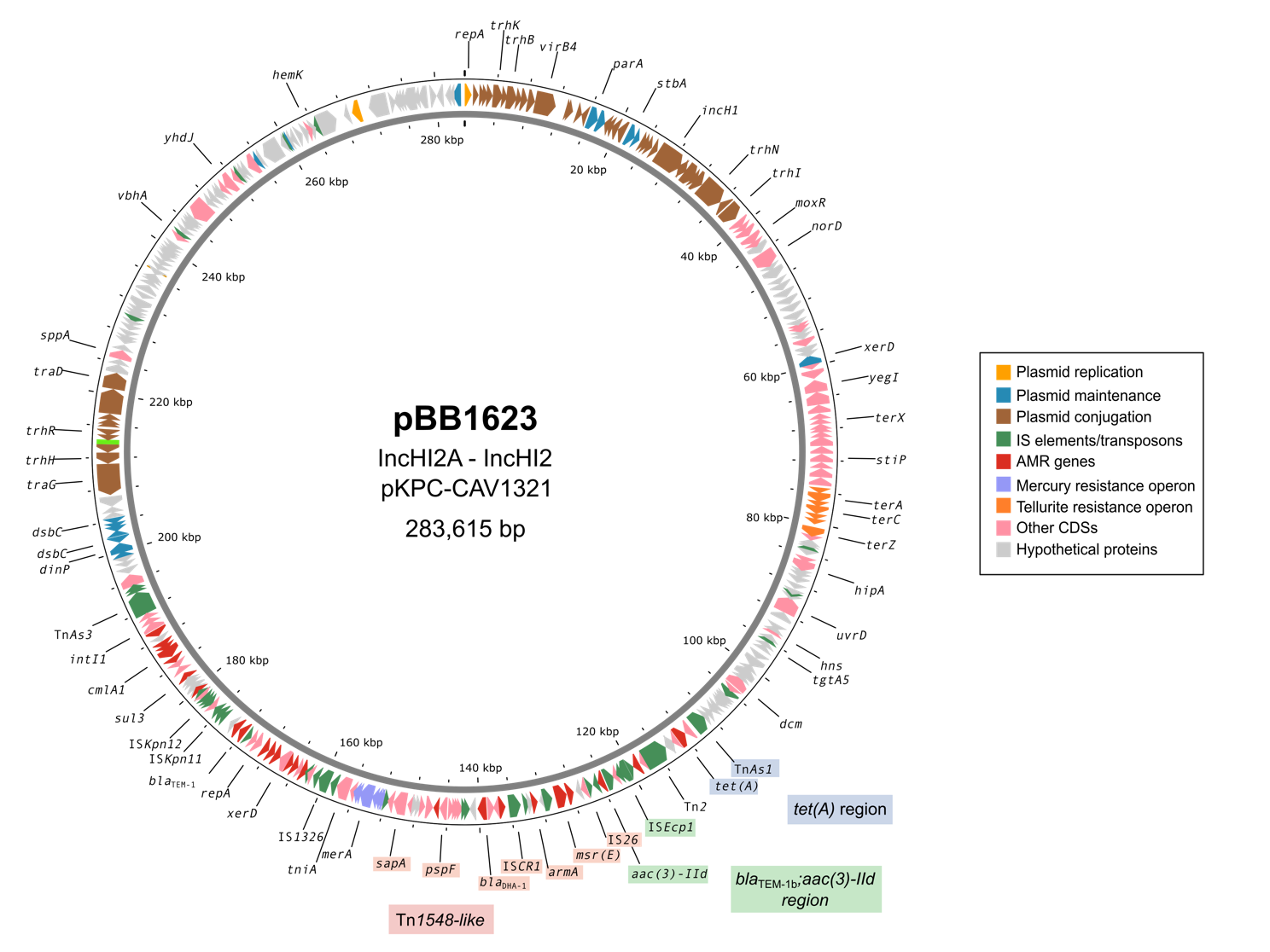
**

**Figure S6.** Genetic map of *Mixta calida* pBB1623 IncHI2A/InHI2/pKPC-CAV1321 plasmid. Genes are denoted as arrows. Plasmid replication genes are depicted in light orange, plasmid maintenance genes in blue, plasmid conjugation genes in brown, IS elements and transposons in green, ARGs in red, the *mer* operon in purple, tellurite resistance operon in dark orange and other coding sequences (CDSs) in pink. Hypothetical proteins are shown in grey. The Tn*1548*-like region is shaded in red, the *tet(A)* region in blue and the *bla*_TEM-1b_;*aac(3)-IId* region in green.
